# Reliability of clinical orthodontic indicators in the Norwegian Registry of Cleft Lip and Palate

**DOI:** 10.1186/s12903-025-07043-6

**Published:** 2025-11-10

**Authors:** Paul K. Saele, Dagrun Slettebø Daltveit, Ragnar Bjering, Tone Klepsland, Nina Ellen Torgersbråten, Christer Kubon, Sissel Gavle, Åse Sivertsen

**Affiliations:** 1 Department of Plastic, Hand and Reconstructive Surgery, Haukeland University Hospital, Bergen, Norway; 2 Norwegian Quality Registry of Cleft Lip and Palate, Surgical Clinic, Haukeland University Hospital, Bergen, Norway; 3 Department of Global Public Health and Primary Care, University of Bergen, Bergen, Norway; 4 Department of Plastic Surgery, Oslo University Hospital, Oslo, Norway; 5 Oral Health Centre of Expertise in Western Norway, Bergen, Norway

**Keywords:** Cleft lip and palate, Quality registry, Reliability

## Abstract

**Background:**

The aim of this study was to assess to the reliability of the orthodontic data for 6-year-old patients with unilateral cleft lip and palate (UCLP) as recorded in the Norwegian Registry of Cleft Lip and Palate (NRCLP).

**Methods:**

This study included 64 children with UCLP, born between 2011 and 2015 and enrolled in the two Norwegian cleft centers. All participants had orthodontic recordings from the age of six in the NRCLP and corresponding dental model casts and radiographs available from the cleft centers. Data on dentoalveolar relations, assessed using GOSLON yardstick, the presence of supernumerary teeth and agenesis, and cephalometric values for intermaxillary sagittal relations recorded in the NRCLP were compared to reassessments conducted by four independent and blinded orthodontists. Intra-rater and inter-rater agreement were evaluated using single measure intraclass correlation coefficients and Gwet’s agreement coefficients.

**Results:**

Comparisons between reassessments and the NRCLP data showed substantial to almost perfect agreement for all orthodontic indicators. Intra-rater agreement for GOSLON was almost perfect. Inter-rater agreement among the four orthodontists was almost perfect for GOSLON scores and for assessment of supernumerary teeth and agenesis, and substantial for the intermaxillary sagittal relations.

**Conclusions:**

This study demonstrates that NRCLP in daily use since 2011, shows robust orthodontic indicators and reliable and trustworthy data for six-year-old children with UCLP.

**Supplementary Information:**

The online version contains supplementary material available at 10.1186/s12903-025-07043-6.

## Background

Norway has a long tradition of centralized multidisciplinary treatment of children born with cleft lip and palate (CLP), with systematic documentation of care from the newborn period to young adulthood [1, 2]. Comprehensive epidemiological studies have been conducted with national data in combination with the Medical Birth Registry of Norway [2–4]. These national studies and the establishment of the Norwegian Registry of Cleft Lip and Palate (NRCLP) in 2011 have been facilitated by a national identification number (since 1964).

The treatment of children born with CLP aims to achieve good facial appearance, normal speech and hearing and good dental occlusion and aesthetics. A multidisciplinary treatment approach from birth to adulthood is important [5, 6]. Surgical treatment of the cleft affects the maxillary growth and makes the patients susceptible to develop a retrognathic midface, negative overjet, and crossbite [7, 8]. Dental anomalies such as agenesis, peg-shaped teeth, and malocclusions are more common findings among children with CLP than without clefts [9–13]. Missing teeth have a negative impact on facial growth [14]. Also, missing lateral incisor and maxillary hypoplasia in children with clefts are predictors for a future need of Le Fort advancement, as described by Lai et al. [15].

In the Eurocleft studies, Semb et al. and Shaw et al. concluded that measurement of the clinical outcome in childhood is an important and valid form of clinical audit and that inter-center studies are more informative than single center reports [16, 17]. Collecting standardized records through the adolescent growth period provides a valuable basis for retrospective studies of treatment outcomes and inter-center comparisons [18]. This motivated the establishment of the NRCLP in 2011. The orthodontists in the Norwegian cleft teams are responsible for monitoring facial and dental development in children born with CLP from birth to adulthood. The orthodontic treatment is carried out at either one of the cleft centers or the team orthodontists organize treatment at a local regional orthodontic clinic. Dental records, radiographs, photos and dental model casts have been systematically collected and archived at the two centers since the sixties [1, 2], and since 2011, orthodontic data are also recorded into the NRCLP at 6-,10- and 16-years of age. The aim of this study is to assess the reliability of the orthodontic data for 6-year-old patients with unilateral cleft lip and palate (UCLP) recorded in the NRCLP.

## Methods

### Norwegian Registry of Cleft Lip and Palate (NRCLP)

NRCLP [19] was established in 2011 by the Norwegian health authorities in cooperation with the two national cleft teams. By 2023, more than 1300 patients born since 2011 with orofacial clefts treated by the two national CLP-teams either in Oslo or in Bergen are included in the NRCLP. The inclusion rate is high, with 95% of parents of children consenting to their child’s participation. Every third-year coverage analyses are comparing registrations in the registry to the recordings in the Norwegian Patient Registry. It has been mandatory by Norwegian Law since 2019 for healthcare professionals to provide data to the NRCLP and this has enhanced the completeness of the registry data. In 2022, the NRCLP database has complete orthodontic data for 90% for all patients at the age of 6 years [20].

The Norwegian cleft teams’ standard orthodontic assessment of a six-year-old child contains photographs, a panoramic radiograph, three intra-oral radiographs of the front segment, a lateral cephalogram and impression for dental plaster models (intraoral digital scanning for models since 2021). All are stored in the clinics. The cleft team orthodontist registers the data electronically into the NRCLP immediately after the assessment. A total of 8 orthodontists have performed orthodontic assessments and registrations into the NRCLP since the start in 2011.

The clinical orthodontic indicators registered in NRCLP at the age of six years are: *sagittal position of the maxilla and mandibula*; *agenesis and supernumerary teeth*; and *dentoalveolar relation*. Registration of the *sagittal position of the maxilla and mandibula* are based on lateral cephalometric analyses. The variables describe the angles between sella turcica, to nasion and respectively subspinale point A (SNA) and supramentale point B (SNB) [21]. The angle that evaluates the anteroposterior relationship between the maxilla and mandible (ANB) is calculated as the difference between the SNA and SNB angles [21]. Registration of *agenesis and supernumerary teeth* is based on intraoral radiographs and orthopantomogram (OPG), with teeth numbering according to ISO 3950 (ISO 3950:2016, Dentistry Designation system for teeth and areas of the oral cavity, 4th edition). Agenesis of 12 and 22 and supernumerary 12 and 22 are specified dummy variables (yes/no), while the two other variables, supernumerary and teeth agenesis are the specified teeth numbers. *Dentoalveolar relation* is recorded by the GOSLON (Great Ormond Street, London and Oslo) yardstick scale [22], which ranks the dental study model in five categories: Excellent relation (1), good relation (2), fair relation (3), poor relation (4) very poor relation (5).

### Participants

All children born with UCLP between 2011 and 2015, and who are included in the NRCLP and have routine orthodontic records stored in the two cleft teams, participated in the study. This resulted in 64 children. The mean age at assessment was 6 years and 0 months (range 5 years and 6 months to 6 years and 11 months). In total, 73% were males and 27% females, a male-to-female ratio which is common in UCLP populations [2].

### Reassessments of dental records and orthodontic documentation

Four experienced and independent cleft orthodontists, two from the team in Oslo and two from the team in Bergen independently assessed the anonymized and blinded dental model casts, orthopantomogram (OPG), lateral cephalogram, and intra-oral radiographs of the 64 patients included. To ensure blinding, the reassessments were performed using paper-printed radiographs, though high-quality digital radiograph were available for the orthodontists at the time of the original registration in the NRCLP. The dental model casts were reassessed 16 h later to evaluate the intra-rater agreement for the GOSLON yardstick measure of dentoalveolar relation. A written instruction of scoring using the GOSLON yardstick scale, and a set-up of dental study cast models for each of the five categories were available to the four orthodontists while scoring. This setup corresponds to the setup in the national centers at the time of first registration into the registry. The repeated assessment was only performed for the GOSLON score because we regarded this score to be more sensitive to subjective evaluations than the other scores.

### Statistical analysis

The sample size for the study was based on the number of individuals that will minimize the error margin of the precent agreement between two arbitrary raters [23]. If the percent agreement lies between 70% and 95%, the required sample size ranges from 28 to 51 individuals, thus our sample size of 64 is appropriate. Intra-rater agreement between the reassessments performed by the four blinded orthodontists was evaluated by calculating percent agreement, Fleiss’ kappa for nominal variables and Fleiss’ ordinal weighted kappa for ordinal variables, and Gwet’s AC_1_ for nominal variables and Gwet’s AC_2_ for ordinal variables [23]. Inter-rater agreement between the reassessments and variables recorded in NRCLP was evaluated by calculating percent agreement, Cohen’s kappa for nominal variables and Cohens’ ordinal weighted kappa for ordinal variables, and Gwet’s AC_1_ for nominal variables and Gwet’s AC_2_ for ordinal variables.

For the continuous variables, inter-rater agreement between the reassessments and inter-rater agreement between the reassessments and variables recorded in the NRCLP was evaluated by absolute inter-judge agreement (individual ICCs) calculated using two-way random-effects models. Finally, intra-rater agreement for the reassessments performed by four independent and blinded orthodontists was evaluated by calculating percent agreement, Cohens’ ordinal weighted kappa for ordinal variables, and Gwet’s AC_2_ for ordinal variables. See Supplementary Table S1 (appendix, p.2) for an overview of the methods used for measuring inter- and intra-rater agreement.

As sensitivity analyses, we made Bland-Altman plots for pairwise comparisons of the reassessments to explore any presence of systematic or proportional biases.

We evaluated the level of chance-corrected agreement based on Gwet’s agreement coefficients [24], according to the criteria proposed by Landis and Koch [25, 26]: values from 0 to 0.20 implies slight agreement, 0.21–0.40 fair, 0.41–0.60 moderate, 0.61–0.80 substantial, and >0.80 almost perfect agreement.

## Results

### Reliability of reassessments

The intra-rater agreement of the four orthodontists’ reassessments of GOSLON yardstick was almost perfect (0.89–0.98) as seen in Table 1.

**Table 1:** Intra-rater agreement for the reassessments for the GOSLON yardstick performed by four independent and blinded orthodontists

Variable	*N*	Percent agreement (95% CI)	Cohens’ κ^a^ (95% CI)	Gwet’s AC_2_ (95% CI)
Goslon yardstick				
Orthodontist 1	64	0.98 (0.97-0.99)	0.86 (0.80-0.93)	0.93 (0.90-0.96)
Orthodontist 2	64	0.99 (0.99-1.00)	0.97 (0.94-1.00)	0.98 (0.96-1.00)
Orthodontist 3	64	0.96 (0.95-0.98)	0.83 (0.75-0.90)	0.89 (0.84-0.93)
Orthodontist 4	64	0.98 (0.97-0.99)	0.90 (0.85-0.96)	0.93 (0.90-0.97)

^a^Cohen’s ordinal weighted kappa. *Abbreviations*: *AC* Agreement Coefficient, *CI* Confidence Interval

The inter-rater agreement (Gwet’s AC) between the reassessments performed by the four independent orthodontists was almost perfect (≥ 0.85) for the Goslon yardstick, agenesis, and supernumerary teeth as presented in Table 2. Agreements were 0.88 for GOSLON yardstick, 0.96 for agenesis of tooth number 12, 0.92 for agenesis of tooth number 22, 0.92 for agenesis of other teeth, 0.97 for supernumerary tooth number 12, 0.90 for supernumerary tooth number 22, and 0.85 for supernumerary other teeth.

**Table 2:** Inter-rater agreement between the reassessments performed by four independent and blinded orthodontists for the orthodontic indicators in terms of Goslon yardstick, agenesis, and supernumerary teeth

Variable	*N*	Percent agreement (%)	Fleiss’ κ^a^ (95% CI)	Gwet’s AC^b^ (95% CI)
Goslon yardstick	64	0.96 (0.95-0.98)	0.81 (0.72-0.91)	0.88 (0.83-0.93)
Agenesis tooth # 12	64	0.97 (0.93-1.00)	0.90 (0.74-1.00)	0.96 (0.91-1.00)
Agenesis tooth # 22	64	0.95 (0.89-1.00)	0.88 (0.72-1.00)	0.92 (0.81-1.00)
Agenesis other teeth	64	0.96 (0.91-1.00)	0.92 (0.83-1.00)	0.92 (0.83-1.00)
Supernumerary tooth # 12	64	0.97 (0.94-1.00)	0.83 (0.62-1.00)	0.97 (0.93-1.00)
Supernumerary tooth # 22	64	0.92 (0.87-0.98)	0.67 (0.41-0.93)	0.90 (0.82-0.98)
Supernumerary other teeth	64	0.87 (0.66-1.00)	0.04 (−0.37-0.44)	0.85 (0.60-1.00)

^a^Fleiss’ kappa for nominal variables and Fleiss’ ordinal weighted kappa for ordinal variables. ^b^Gwet’s AC_1_ for nominal variables and Gwet’s AC_2_ for ordinal variables. *Abbreviations*: *AC* Agreement Coefficient

Table 3 depicts the agreement (ICCs) between the four orthodontists in the reassessments and was almost perfect for SNA and SNB (0.81 and 0.89, respectively), and substantial for the ANB angulation (0.71).

**Table 3:** Absolute inter-judge agreement (individual ICCs) was calculated using two-way random-effects models for sagittal relation (facial profile) measured from lateral cephalogram in terms of SNA, SNB, and ANB angulations

Variable	*N*	ICC (95% CI)
SNA angle	64	0.81 (0.70-0.88)
SNB angle	64	0.89 (0.84-0.93)
ANB angle	64	0.71 (0.56-0.82)

*Abbreviations*: *ANB* the anteroposterior relationship between the maxilla and mandible, *ICC* intraclass correlation coefficient, *SNA* the subspinale point A, *SNB* the supramentale point B

### Comparison of registry data and reassessments

Inter-rater agreement between the reassessments and variables recorded in the NRCLP was conducted for each orthodontist separately as shown in Table 4. The agreement for GOSLON yardstick was substantial to almost perfect for all four orthodontists (0.75–0.81). For agenesis of teeth numbers 12 and 22, and agenesis of other teeth, and for supernumerary teeth numbers 12 and 22, the agreements were almost perfect for all four orthodontists (0.88–1.0.88.0). The agreement for the variable “supernumerary other teeth” was substantial (0.77) for one of the orthodontists, and almost perfect for the other three raters (0.97–0.98).

**Table 4:** Inter-rater agreement between the reassessments and variables recorded in the Norwegian registry of cleft-lip and palate registry performed by four independent and blinded orthodontists

Variable	*N*	Percent agreement (95% CI)	Cohen’s κ^a^ (95% CI)	Gwet’s AC^b^ (95% CI)
Goslon yardstick				
Orthodontist 1	64	(0.87-1.00)	0.64 (0.47-0.81)	0.81 (0.71-0.92)
Orthodontist 2	64	0.92 (0.85-0.99)	0.58 (0.40-0.76)	0.76 (0.64-0.88)
Orthodontist 3	64	(0.87-1.00)	0.60 (0.44-0.77)	0.80 (0.70-0.90)
Orthodontist 4	64	0.92 (0.85-0.99)	0.57 (0.40-0.74)	0.75 (0.63-0.86)
Agenesis tooth # 12				
Orthodontist 1	64	1.00 (1.00-1.00)	1.00 (1.00-1.00)	1.00 (1.00-1.00)
Orthodontist 2	64	(0.95-1.00)	0.94 (0.82-1.00)	(0.94-1.00)
Orthodontist 3	64	(0.88-1.00)	0.83 (0.63-1.00)	(0.84-1.00)
Orthodontist 4	64	1.00 (1.00-1.00)	1.00 (1.00-1.00)	1.00 (1.00-1.00)
Agenesis tooth # 22				
Orthodontist 1	64	(0.91-1.00)	(0.81-1.00)	(0.86-1.00)
Orthodontist 2	64	(0.88-1.00)	(0.75-1.00)	(0.81-1.00)
Orthodontist 3	64	(0.90-1.00)	(0.81-1.00)	(0.85-1.00)
Orthodontist 4	64	(0.87-1.00)	(0.70-1.00)	(0.78-1.00)
Agenesis other teeth				
Orthodontist 1	64	(0.84-1.00)	(0.72-1.00)	(0.73-1.00)
Orthodontist 2	64	(0.87-1.00)	(0.77-1.00)	(0.77-1.00)
Orthodontist 3	64	(0.87-1.00)	(0.76-1.00)	(0.78-1.00)
Orthodontist 4	64	(0.87-1.00)	(0.77-1.00)	(0.77-1.00)
Supernumerary tooth # 12				
Orthodontist 1	64	(0.94-1.00)	(0.67-1.00)	(0.93-1.00)
Orthodontist 2	64	(0.91-1.00)	(0.53-1.00)	(0.90-1.00)
Orthodontist 3	64	(0.94-1.00)	(0.67-1.00)	(0.93-1.00)
Orthodontist 4	64	(0.97-1.00)	(0.97-1.00)	(0.97-1.00)
Supernumerary tooth # 22				
Orthodontist 1	64	0.91 (0.83-0.98)	0.57 (0.26-0.89)	0.88 (0.78-0.98)
Orthodontist 2	64	(0.91-1.00)	(0.67-1.00)	(0.89-1.00)
Orthodontist 3	64	0.91 (0.83-0.98)	0.52 (0.18-0.86)	0.88 (0.78-0.98)
Orthodontist 4	64	(0.88-1.00)	0.74 (0.50-0.99)	(0.83-1.00)
Supernumerary other teeth				
Orthodontist 1	64	(0.95-1.00)	(0.02-1.00)	(0.95-1.00)
Orthodontist 2	64	0.81 (0.71-0.91)	0.12 (−0.10-0.33)	0.77 (0.62-0.91)
Orthodontist 3	64	(0.95-1.00)	0.00 (0.00-0.00)	(0.95-1.00)
Orthodontist 4	64	(0.76-1.00)	0.60 (−0.10-1.00)	(0.76-1.00)

^a^Cohen’s kappa for nominal variables and Cohens’ ordinal weighted kappa for ordinal variables. ^b^Gwet’s AC_1_ for nominal variables and Gwet’s AC_2_ for ordinal variables. *Abbreviations*: *AC* Agreement Coefficient

Inter-rater agreement (individual ICCs) between the registry and the reassessments of SNA and SNB showed substantial to almost perfect (0.76–0.92) agreement for all four orthodontists (Table 5). Agreement for the ANB angle varied from substantial to almost perfect (0.62–0.83).

**Table 5:** Absolute inter-judge agreement (individual ICCs) calculated using two-way random-effects models for intermaxillary sagittal relation (facial profile) measured from lateral cephalogram in terms of SNA, SNB and ANB angulations

Variable	*N*	ICC (95% CI)
SNA angulation		
Orthodontist 1	63	0.83 (0.73-0.89)
Orthodontist 2	63	0.76 (0.63-0.85)
Orthodontist 3	63	0.77 (0.51-0.88)
Orthodontist 4	63	0.83 (0.73-0.89)
SNB angulation		
Orthodontist 1	63	0.88 (0.76-0.94)
Orthodontist 2	63	0.78 (0.55-0.89)
Orthodontist 3	63	0.92 (0.87-0.95)
Orthodontist 4	63	0.86 (0.75-0.92)
ANB angulation		
Orthodontist 1	63	0.82 (0.72-0.89)
Orthodontist 2	63	0.62 (0.12-0.83)
Orthodontist 3	63	0.65 (0.09-0.85)
Orthodontist 4	63	0.83 (0.72-0.89)

*Abbreviations*: *ANB* the anteroposterior relationship between the maxilla and mandible, *ICC* intraclass correlation coefficient, *SNA* the subspinale point A, *SNB* the supramentale point B

Bland-Altman plots of pairwise comparisons of the reassessments did not suggest any presence of systematic or proportional biases in our study (Supplementary Figures S1–S6).

## Discussion

This study examined the clinical orthodontic variables recoded in NRCLP for six-year-old children with UCLP born between 2011 and 2015. The results demonstrated substantial to almost perfect intra- and inter-rater reliability for all orthodontic variables.

The value of registry data is highly dependent on the validity of the clinical indicators, completeness of the registrations and reliability of the data. Systematic evaluation of the reliability of collected data, such as this study in NRCLP, is essential. Variables in a national cleft registry must be well defined, easy to measure, and not affected by subjective variations. The high level of agreement between data registered by eight different orthodontists in NRCLP and the reassessment years later by four colleagues demonstrates a shared understanding of the variable definitions among the scorers. When the registry was established in 2011, the scoring by orthodontist from the two national cleft centers was calibrated, and this calibration has been maintained through annual meetings. The common understanding might also have been kept because the variables are clinically relevant in the orthodontists’ day-to day treatment of CLP patients. Additionally, a well-structured technical solution for electronic registration to the registry, with mandatory validation fields, and clear explanations of the variables within the electronic forms, may have facilitated consistent data registration.

The GOSLON yardstick is the most commonly used index for evaluation of occlusal outcome and severity of crossbite in children with UCLP in the late mixed/early permanent dentition [27]. It has been used by the Norwegian cleft teams since the start of the Scandcleft studies in 2001 [28] at the ages 6 and 10 years. Other commonly used similar indices are the 5YO index [29, 30], Eurocran index [31], the Huddart/Bodenham (HB) index [32] in deciduous dentition, and the Modified Huddart and Bodenham (MHB) index in mixed and permanent dentitions [33]. They all categorize occlusion by assessing anteroposterior, vertical, and transversal relationships. The Swedish Quality Registry for Cleft Lip and Palate [34] included the 5YO and MHB indexes in its variables in 2016. Good agreement among the Swedish cleft teams assessing the 5YO and MHB indexes and good inter-rater and intra-rater agreement were reported for both indexes in a reassessment of 61 patients by 14 orthodontists [35]. The Cleft Registry and Audit Network (CRANE) [36] in Great Britain uses the 5YO index to assess facial growth at the age of five. To ensure standardization in scoring, external scores by a blinded process undertaken by calibrated examiners have been established. Obtaining complete cohorts is a challenge with this system of data collection, as just under 50% of the eligible children had their 5YO index recorded in the latest annual reports [37]. In the Scancleft study, reliability of the Eurocran index, 5YO and GOSLON Yardstick were deemed acceptable, with significant correlations between the dental indexes at all ages [28, 38]. A similarly high level of repeatability of GOSLON Yardstick has been shown in other clinical cohort studies [35, 39, 40].

Cephalometric measurements have been deemed highly reliable in clinical cohort studies [41–43] and the SNA, SNB, and ANB measurements as registered in NRCLP have been validated for use in international studies, such as Scandcleft [38] and Eurocleft [44]. However, as far as we know, our study is the first to present the reliability of recordings related to cephalometric measurements of intermaxillary sagittal relations and to supernumerary teeth and agenesis, in data that are prospectively reported in a national quality registry.

Paper-printed radiographs at the time of reassessments may have made the scoring more difficult, in particular when the images were blurry, compared to at the time of registration in the NRCLP when high-quality digital radiographs were available. This does not seem to have affected the accuracy and reliability of the variables to a significant degree. However, it is possible that measuring the intermaxillary sagittal angles is more difficult using paper printed copies compared to software, and similarly for counting supernumerary teeth. This could explain that the agreement for ANB angulation was only substantial. This could also have affected the agreement between registry data and raters for the variable “supernumerary other teeth” which varied from substantial (one rater) to almost perfect (three raters).

Many cleft centers maintain local clinical registries to evaluate, understand and enhance their performance. Comparing data and clinical results between these cleft centers is invaluable for improving cleft care. As a result, some treatment centers have established quality registries that include several hospitals, such as CORNET Utah [45], while others collaborate on a national level, such as the Swedish Registry of Cleft Lip and Palate or CRANE. Additionally international clinical networks, including European Reference Networks Cranio [46], Operation Smile [47], Smile Train [48], work on development of registries for cleft treatment across national borders. Extensive work is required to select valid variables, ensure compliance with general data protection regulations, secure financial funding, and provide IT- support to establish accurate and sustainable registration by the clinicians. Ultimately, complete and reliable data are essential. The information recorded must be based on a uniform understanding of the variables among the clinicians reporting it, and the results must be possible to reproduce. Our study highlights the essential oversight that all registries must implement regularly to ensure high data quality.

## Conclusions

This national cleft registry in daily use for over twelve years shows robust orthodontic indicators and reliable and trustworthy data. We conclude that the GOSLON Yardstick scale, the number of agenesis and supernumerary teeth, SNA, SNB, and ANB can be considered reliable measures in NRCLP for six-year-old children with UCLP.

## Supplementary Information

Supplementary Material 1.

## Data Availability

The datasets generated and analyzed during the current study are not publicly available due to data privacy laws but are available from the corresponding author on reasonable request.

## Abbreviations

NRCLP: Norwegian Registry of Cleft Lip and Palate
UCLP: Unilateral cleft lip and palate
GOSLON: Great Ormond Street, London and Oslo
CLP: Cleft lip and palate
SNA: Subspinale point A
SNB: Supramentale point B
ANB: Anteroposterior relationship between the maxilla and mandible
OPG: Orthopantomograph
HB index: Huddart/Bodenham index
MHB index: Modified Huddart and Bodenham index
CRANE: Cleft Registry and Audit Network
