# Supplementary material for: Reliability of clinical orthodontic indicators in the Norwegian Registry of Cleft Lip and Palate
Source: BMC Oral Health. 2025 Nov 10;25:1763. doi: 10.1186/s12903-025-07043-6 (PMC12599112; doi:10.1186/s12903-025-07043-6)
Supplement: Supplementary file 1 — Supplementary Material 1. [file 12903_2025_7043_MOESM1_ESM.docx]

Supplementary Materials to

**Reliability of clinical orthodontic indicators in the Norwegian Registry of Cleft Lip and Palate**

Paul K. Saele^1^, Dagrun Slettebø Daltveit^2,3^, Ragnar Bjering^4^, Tone Klepsland^5^, Nina Ellen Torgersbråten^4^, Christer Kubon^1,2^, Sissel Gavle^1^, Åse Sivertsen^1,2^

^1^Department of Plastic, Hand and Reconstructive Surgery, Haukeland University Hospital, Bergen, Norway. ^2^Norwegian Quality Registry of Cleft Lip and Palate, Surgical Clinic, Haukeland University Hospital, Bergen, Norway. ^3^University of Bergen, Norway. ^4^Oslo University Hospital, Oslo, Norway. ^5^Oral Health Centre of Expertise/Western Norway.

Table of Content

[Table S1. An overview of the methods used for measuring inter- and intra-rater agreement. 2](#_Toc199340040)

[Figure S1. SNA rating differences as a function of rating means for the reassessments done by four different raters. 3](#_Toc199340041)

[Figure S2. SNB rating differences as a function of rating means for the reassessments done by four different raters. 3](#_Toc199340042)

[Figure S3. ANB rating differences as a function of rating means for the reassessments done by four different raters. 4](#_Toc199340043)

[Figure S4. SNA rating differences as a function of rating means for the registry data versus reassessments done by four different raters. 4](#_Toc199340044)

[Figure S5. SNB rating differences as a function of rating means for the registry data versus reassessments done by four different raters. 5](#_Toc199340045)

[Figure S6. ANB rating differences as a function of rating means for the registry data versus reassessments done by four different raters. 5](#_Toc199340046)

# Table S1. An overview of the methods used for measuring inter- and intra-rater agreement.

| **Variable** | **Type** | **Number of measurements** | **Methods** |
| --- | --- | --- | --- |
| *Inter-rater agreement between the reassessments of the four orthodontists* | | | |
| Goslon yardstick | Ordinal | 4 | Percent agreement, Fleiss’ ordinal weighted kappa, and Gwet’s AC_2_. |
| Agenesis tooth # 12 | Nominal | 4 | Percent agreement, Fleiss’ kappa, and Gwet’s AC_1_. |
| Agenesis tooth # 22 | Nominal | 4 | Percent agreement, Fleiss’ kappa, and Gwet’s AC_1_. |
| Agenesis other teeth | Nominal | 4 | Percent agreement, Fleiss’ kappa, and Gwet’s AC_1_. |
| Supernumerary tooth # 12 | Nominal | 4 | Percent agreement, Fleiss’ kappa, and Gwet’s AC_1_. |
| Supernumerary tooth # 22 | Nominal | 4 | Percent agreement, Fleiss’ kappa, and Gwet’s AC_1_. |
| Supernumerary other teeth | Nominal | 4 | Percent agreement, Fleiss’ kappa, and Gwet’s AC_1_. |
| SNA angle | Continuous | 4 | ICC. |
| SNB angle | Continuous | 4 | ICC. |
| ANB angle | Continuous | 4 | ICC. |
| *Inter-rater agreement between the reassessments and values recorded in the NRCL* | | | |
| Goslon yardstick | Ordinal | 2 | Percent agreement, Cohens’ ordinal weighted kappa, and Gwet’s AC_2_. |
| Agenesis tooth # 12 | Nominal | 2 | Percent agreement, Cohen’s kappa, and Gwet’s AC_1_. |
| Agenesis tooth # 22 | Nominal | 2 | Percent agreement, Cohen’s kappa, and Gwet’s AC_1_. |
| Agenesis other teeth | Nominal | 2 | Percent agreement, Cohen’s kappa, and Gwet’s AC_1_. |
| Supernumerary tooth # 12 | Nominal | 2 | Percent agreement, Cohen’s kappa, and Gwet’s AC_1_. |
| Supernumerary tooth # 22 | Nominal | 2 | Percent agreement, Cohen’s kappa, and Gwet’s AC_1_. |
| Supernumerary other teeth | Nominal | 2 | Percent agreement, Cohen’s kappa, and Gwet’s AC_1_. |
| SNA angle | Continuous | 2 | ICC. |
| SNB angle | Continuous | 2 | ICC. |
| ANB angle | Continuous | 2 | ICC. |
| *Intra-rater agreement for the reassessments performed by the four orthodontists* | | | |
| Goslon yardstick | Ordinal | 2 | Percent agreement, Cohen’s ordinal weighted kappa, and Gwet’s AC_2_. |

# Figure S1. SNA rating differences as a function of rating means for the reassessments done by four different raters.

# Figure S2. SNB rating differences as a function of rating means for the reassessments done by four different raters.

# Figure S3. ANB rating differences as a function of rating means for the reassessments done by four different raters.

# Figure S4. SNA rating differences as a function of rating means for the registry data versus reassessments done by four different raters.

# Figure S5. SNB rating differences as a function of rating means for the registry data versus reassessments done by four different raters.

# Figure S6. ANB rating differences as a function of rating means for the registry data versus reassessments done by four different raters.
